# HEWMA-based memory type estimator for ranked set sampling with two auxiliary variables: application to cancer mortality data

**DOI:** 10.1038/s41598-026-50886-4

**Published:** 2026-05-02

**Authors:** Eda Gizem Koçyiğit

**Affiliations:** Department of Statistics, Dokuz Eylül University, 35390 Buca, İzmir, Türkiye

**Keywords:** Computational science, Statistics

## Abstract

Ranked Set Sampling (RSS) is known for its efficiency in parameter estimation, especially when ranking is more feasible than actual measurement. This study introduces a novel memory type estimator for RSS based on Hybrid Exponentially Weighted Moving Averages (HEWMA), using two auxiliary variables. The estimator aims to enhance efficiency by integrating current and past information along with secondary auxiliary data. Analytical expressions for the bias and mean square error (MSE) are derived, and the corresponding sample coefficients are obtained to simplify HEWMA weight calculations. A simulation study is conducted to evaluate the estimator under various conditions, including different sample sizes, distributional shapes (normal and skewed Weibull), and varying correlation levels among study and auxiliary variables. Results indicate that the proposed estimator consistently yields the highest relative efficiency (RE) compared to conventional and memory estimators using one or multiple auxiliary variables. Additionally, the estimator is applied to two real-world mortality datasets from the USA, involving deaths from tobacco- and alcohol-related cancers. Despite challenges such as zero values and small sample sizes, the estimator maintains superior performance. Overall, the proposed estimator offers an improvement in estimation performance for RSS, particularly in settings where auxiliary variables are available and memory type estimators are applicable.

**Supplementary Information:**

The online version contains supplementary material available at 10.1038/s41598-026-50886-4.

## Introduction

Ranked Set Sampling (RSS) is recognized for providing more cost-effective, time-efficient, and accurate results compared to Simple Random Sampling (SRS)^1,2^. RSS relies on auxiliary variables that are relatively easy to observe and strongly correlated with the study variable. Such variables play a central role in the ranking process and sample selection, substantially enhancing the efficiency of the sampling scheme.

RSS can be utilized during the estimation phase through various estimators, including ratio, product, or regression-based methods, to further improve precision^3–7^. In recent years, considerable attention has been devoted to developing improved estimators and statistical procedures under the RSS framework. Various studies have investigated parameter estimation and distributional properties under RSS schemes, demonstrating their advantages over conventional sampling methods. For example, recent work has examined maximum likelihood estimation and the parameter properties of specific distributions under the RSS design^8^. Other studies have explored missing data imputation using multiple auxiliary variables^9^, logarithmic estimators for small-area estimation^10^, and enhancements to logistic regression classification^11^, highlighting the versatility and efficiency of RSS.

To enhance the efficiency of statistical estimators, researchers have proposed incorporating known population parameters when available^12–14^. Using multiple auxiliary variables has been shown to yield greater efficiency than relying on a single variable. Several studies in^15–20^ have developed estimators that incorporate multiple auxiliary variables and have demonstrated improved performance compared to classical single-variable approaches across various sampling settings. Auxiliary information has also been effectively utilized for estimating different population parameters beyond the mean. For instance, a family of estimators has been proposed for estimating the population median using auxiliary information, further highlighting the flexibility and importance of such approaches in survey sampling^21^. These findings emphasize the importance of utilizing additional auxiliary information to achieve higher estimation accuracy.

Another line of research emphasizes the benefit of combining past and current sample information to improve estimation. These approaches often employ Exponentially Weighted Moving Average (EWMA) or Hybrid EWMA (HEWMA) control chart statistics. Past sample means have been incorporated into exponential ratio and regression-type estimators under SRS to improve estimation efficiency^22^, while memory-type ratio and product estimators were proposed for time-based surveys, highlighting the usefulness of incorporating historical information into the estimation process^23^. A family of EWMA-based memory type estimators for the estimation of population mean is proposed under SRS^24^. The performance of logarithmic memory-type estimators under SRS was evaluated, showing efficiency improvements over traditional approaches^25^. Further developments include combining memory-type ratio and product estimators within an extended EWMA statistic with applications to real data problems such as wheat production^26^, as well as new memory-based ratio estimators designed to enhance estimation accuracy using past information^27^. Variance estimation based on EWMA-type memory estimators was also studied for time-scaled surveys in stratified sampling^28^, and optimal classes of memory-type imputation methods based on EWMA statistics were developed for time-based survey data^29^. Improved memory-type ratio estimators were proposed for stratified random sampling under different cost structures^30^, and optimal classes of memory-type estimators for temporal surveys were introduced, further demonstrating the effectiveness of EWMA-based approaches^31^. Recent studies have further extended the use of memory-type estimators by incorporating alternative forms of auxiliary information. A generalized class of EWMA-based estimators utilizing binary auxiliary attributes has been proposed, demonstrating improved estimation efficiency, particularly when quantitative auxiliary information is limited^32^. An EWMA-based memory-type estimator using two auxiliary variables was developed for the SRS framework^33^, integrating two distinct strategies: the use of multiple auxiliary variables and memory-type estimation.

Memory-type estimators, including EWMA, HEWMA, and HEEWMA-based approaches, generally rely on assumptions such as the sequential ordering of observations, independence of sampling units, and, in some cases, distributional assumptions such as normality and constant variance. In the context of RSS, the ordering of observations and independence among sets are inherently satisfied by the design, though distributional assumptions may still influence the efficiency and robustness of the estimators. Proper selection of weight parameters is also crucial, as it determines the relative influence of past observations on current estimates. While EWMA incorporates past information through a single smoothing parameter, the HEWMA statistic extends this idea by combining two smoothing mechanisms^34^, allowing a more flexible integration of historical and current information.

In light of these developments, there is a growing need for more flexible and efficient estimation techniques that can simultaneously exploit auxiliary information and incorporate temporal dependence within the sampling framework. While existing studies have addressed these aspects separately, their combined effect within the RSS framework remains relatively underexplored. This motivates further investigation into advanced estimation strategies that can enhance both theoretical efficiency and practical applicability.

## Research gap and contributions

Despite the extensive literature on RSS-based estimators and memory-type estimation techniques, existing studies primarily focus on either single auxiliary variables or memory-type estimators developed under the SRS framework. Although recent work has combined multiple auxiliary variables with memory structures, these approaches have not been extended to the RSS framework using more flexible smoothing mechanisms such as HEWMA. Therefore, a clear gap exists in developing an estimator that simultaneously integrates (i) the efficiency of RSS, (ii) the use of multiple auxiliary variables, and (iii) the flexibility of HEWMA-based memory structures.

Motivated by this gap, the present study proposes a novel HEWMA-based memory-type estimator for the population mean under the RSS framework using two auxiliary variables. The proposed approach integrates hybrid exponential smoothing with auxiliary-variable-based estimation, offering a more flexible and comprehensive framework than traditional EWMA-based estimators. The theoretical properties of the estimator, including bias and mean square error expressions, are derived to establish its statistical validity. Furthermore, the performance of the proposed estimator is systematically evaluated through extensive simulation studies under various distributional settings and correlation structures. Finally, the method’s practical applicability is demonstrated using real-world cancer mortality data, highlighting its effectiveness in real world sampling scenarios.

## Mean estimators in RSS

### Notation

The following key notations and parameters are used throughout the paper:

- Γ: Study variable
- N: First auxiliary variable
- Z: Second auxiliary variable
- $\overline{\Gamma } ,\overline{\rm N} ,\,\,\overline{\rm Z}$: Population means of the study variable Γ, auxiliary variables N and Z, respectively
- $\widehat{\gamma}_{\eta } ,\,\,\widehat{\gamma}_{\zeta }$: RSS sample means of the auxiliary variables N and Z, respectively
- $\gamma_{\left( i \right)}$,$\gamma_{\eta \left( i \right)}$,$\gamma_{\zeta \left( i \right)}$: The *i*-th order statistics of Γ, N and Z
- $\sigma_{\Gamma }^{2}$,$\sigma_{\rm N}^{2}$,$\sigma_{\rm Z}^{2}$ : The population variances of the variables
- $\sigma_{{\Gamma {\rm N}}}$,$\sigma_{{\Gamma {\rm Z}}}$,$\sigma_{{{\rm N}{\rm Z}}}$ : The population covariances between the variables Γ, N and Z
- *m*, *c* : Set size and number of repetitions for RSS
- *ω*_E,_ *ω*_H_ : Weights for EWMA and HEWMA respectively
- $V_{\Gamma }^{2} ,\,V_{\rm N}^{2} ,\,V_{\rm Z}^{2}$: Variances of the variables under RSS with HEWMA coefficient
- $V_{{\Gamma {\rm N}}} ,\,V_{{\Gamma {\rm Z}}} ,V_{NZ}$: Covariances of the variables under RSS with HEWMA coefficient

### Estimators with a single auxiliary variable

The first estimator considered in this study is the RSS simple mean estimator, which is given in Eq. (1).

1 $$\widehat{\gamma}_{1} = \frac{{\sum\limits_{j = 1}^{c} {\sum\limits_{i = 1}^{m} {\gamma_{{\left( {i;j} \right)}} } } }}{mc}$$

The second estimator is the RSS ratio estimator, which incorporates auxiliary information through the ratio of Γ to N. This estimator is defined in Eq. (2) and has been shown to be more efficient when Γ and N are positively correlated^35,36^.

2 $$\widehat{\gamma}_{1 - Rat} = \left( {{{\widehat{\gamma}_{1} } \mathord{\left/ {\vphantom {{\widehat{\gamma}_{1} } {\widehat{\gamma}_{\eta } }}} \right. \kern-0pt} {\widehat{\gamma}_{\eta } }}} \right)\overline{\rm N}$$

The third estimator, the RSS exponential ratio estimator, extends the ratio approach by applying an exponential adjustment. Its mathematical expression is shown in Eq. (3)^37^.

3 $$\widehat{\gamma}_{1 - Exp} = \widehat{\gamma}_{1} \exp \left( {\frac{{\overline{\rm N} - \widehat{\gamma}_{\eta } }}{{\overline{\rm N} + \widehat{\gamma}_{\eta } }}} \right)$$

Lastly, the RSS regression estimator uses a linear regression model to incorporate auxiliary variable information, aiming to reduce estimation variance. This estimator is presented in Eq. (4)^38^.

4 $$\widehat{\gamma}_{{1 - {\mathrm{Re}} g}} = \widehat{\gamma}_{1} + \widetilde{b}_{1} \left( {\overline{\rm N} - \widehat{\gamma}_{\eta } } \right)$$

The parameter $\widetilde{b}_{1}$ represents the regression slope between Γ and N, which can be estimated from the RSS sample.

### Estimators with multiple auxiliary variables

The RSS ratio estimator, exponential ratio estimator, and regression estimator, each involving two auxiliary variables, are presented in Eqs. (5)–(6), respectively.

5 $$\widehat{\gamma}_{2 - Rat} = \widehat{\gamma}_{1} \left( {{{\overline{\rm N} } \mathord{\left/ {\vphantom {{\overline{\rm N} } {\widehat{\gamma}_{\eta } }}} \right. \kern-0pt} {\widehat{\gamma}_{\eta } }}} \right)\left( {{{\overline{\rm Z} } \mathord{\left/ {\vphantom {{\overline{\rm Z} } {\widehat{\gamma}_{\zeta } }}} \right. \kern-0pt} {\widehat{\gamma}_{\zeta } }}} \right)$$

6 $$\widehat{\gamma}_{2 - Exp} = \widehat{\gamma}_{1} \exp \left( {\frac{{\overline{\rm N} - \widehat{\gamma}_{\eta } }}{{\overline{\rm N} + \widehat{\gamma}_{\eta } }}} \right)\exp \left( {\frac{{\overline{\rm Z} - \widehat{\gamma}_{\zeta } }}{{\overline{\rm Z} + \widehat{\gamma}_{\zeta } }}} \right)$$

7 $$\widehat{\gamma}_{{2 - {\mathrm{Re}} g}} = \widehat{\gamma}_{1} + \widetilde{b}_{1} \left( {\overline{\rm N} - \widehat{\gamma}_{\eta } } \right) + \widetilde{b}_{2} \left( {\overline{\rm Z} - \widehat{\gamma}_{\zeta } } \right)$$

Here, $\widetilde{b}_{2}$ indicates the regression slope between Γ and Ζ.

### Memory type estimator

The only EWMA-based memory-type estimator for RSS available in the literature is given by the following equation^39^.

8 $$\widehat{\gamma}_{1 - EW} = \left( {{{\widehat{\gamma}_{E\Gamma \left( T \right)} } \mathord{\left/ {\vphantom {{\widehat{\gamma}_{E\Gamma \left( T \right)} } {\widehat{\gamma}_{{E{\rm N}\left( T \right)}} }}} \right. \kern-0pt} {\widehat{\gamma}_{{E{\rm N}\left( T \right)}} }}} \right)\overline{\rm N}$$

where

$$\widehat{\gamma}_{E\Gamma \left( T \right)} = \omega_{E} \widehat{\gamma}_{1\left( T \right)} + \left( {1 - \omega_{E} } \right)\widehat{\gamma}_{E\Gamma \left( T \right)}$$

and

$$\widehat{\gamma}_{{E{\rm N}\left( T \right)}} = \omega_{E} \widehat{\gamma}_{\eta \left( T \right)} + \left( {1 - \omega_{E} } \right)\widehat{\gamma}_{{E{\rm N}\left( T \right)}}$$

represent the EWMA statistics for Γ and N, respectively, and the weight parameter is defined as *ω*_E_ ∊ (0, 1].

### Proposed estimator

Motivated by estimators proposed in the literature^22,23,33^, we propose an estimator for the RSS method that combines both past and current sample means using two auxiliary variables. The estimator is given by the following equation:

9 $$\widehat{\gamma}_{2 - HEW} = \widehat{\gamma}_{H\Gamma \left( T \right)} \exp \left[ {\frac{{\overline{\rm N} - \widehat{\gamma}_{{H{\rm N}\left( T \right)}} }}{{\overline{\rm N} + \widehat{\gamma}_{{H{\rm N}\left( T \right)}} }}} \right]\exp \left[ {\frac{{\overline{\rm Z} - \widehat{\gamma}_{{H{\rm Z}\left( T \right)}} }}{{\overline{\rm Z} + \widehat{\gamma}_{{H{\rm Z}\left( T \right)}} }}} \right]$$

where

$$\widehat{\gamma}_{H\Gamma \left( T \right)} = \left( {1 - \omega_{H} } \right)\widehat{\gamma}_{{H\Gamma \left( {T - 1} \right)}} + \omega_{H} \widehat{\gamma}_{E\Gamma \left( T \right)},$$

$$\widehat{\gamma}_{{H{\rm N}\left( T \right)}} = \left( {1 - \omega_{H} } \right)\widehat{\gamma}_{{H{\rm N}\left( {T - 1} \right)}} + \omega_{H} \widehat{\gamma}_{{E{\rm N}\left( T \right)}}$$

$$\widehat{\gamma}_{{H{\rm Z}\left( T \right)}} = \left( {1 - \omega_{H} } \right)\widehat{\gamma}_{{H{\rm Z}\left( {T - 1} \right)}} + \omega_{H} \widehat{\gamma}_{{E{\rm Z}\left( T \right)}}$$

 represent the HEWMA statistics for Γ, N and Z, respectively;

$$\widehat{\gamma}_{{E{\rm Z}\left( T \right)}} = \omega_{E} \widehat{\gamma}_{\zeta \left( T \right)} + \left( {1 - \omega_{E} } \right)\widehat{\gamma}_{{E{\rm Z}\left( T \right)}}$$

denotes the EWMA statistic for Z and the weight parameter of HEWMA is defined as *ω*_*H*_ ∊ (0, 1]. The derivation of the bias and MSE of the proposed estimator is presented below using a second-order Taylor series approximation.

To derive the expressions for bias and MSE, the following notations are defined:

$$e_{\Gamma } = {{\left( {\widehat{\gamma}_{H\Gamma \left( T \right)} - \overline{\Gamma } } \right)} \mathord{\left/ {\vphantom {{\left( {\widehat{\gamma}_{H\Gamma \left( T \right)} - \overline{\Gamma } } \right)} {\overline{\Gamma } }}} \right. \kern-0pt} {\overline{\Gamma } }},\,e_{N} = {{\left( {\widehat{\gamma}_{HN\left( T \right)} - \overline{N} } \right)} \mathord{\left/ {\vphantom {{\left( {\widehat{\gamma}_{HN\left( T \right)} - \overline{N} } \right)} {\overline{N} }}} \right. \kern-0pt} {\overline{N} }},$$

and

$$e_{Z} = {{\left( {\widehat{\gamma}_{HZ\left( T \right)} - \overline{Z} } \right)} \mathord{\left/ {\vphantom {{\left( {\widehat{\gamma}_{HZ\left( T \right)} - \overline{Z} } \right)} {\overline{Z} }}} \right. \kern-0pt} {\overline{Z} }}.$$

 Here

$$E\left( {e_{\Gamma } } \right) = E\left( {e_{N} } \right) = E\left( {e_{Z} } \right) = 0,\,E\left( {e_{\Gamma }^{2} } \right) = {{V_{\Gamma }^{2} } \mathord{\left/ {\vphantom {{V_{\Gamma }^{2} } {\overline{\Gamma } }}} \right. \kern-0pt} {\overline{\Gamma } }}^{2},$$

 

$$E\left( {e_{N}^{2} } \right) = {{V_{N}^{2} } \mathord{\left/ {\vphantom {{V_{N}^{2} } {\overline{N} }}} \right. \kern-0pt} {\overline{N} }}^{2},\,E\left( {e_{Z}^{2} } \right) = {{V_{Z}^{2} } \mathord{\left/ {\vphantom {{V_{Z}^{2} } {\overline{Z} }}} \right. \kern-0pt} {\overline{Z} }}^{2}\,E\left( {e_{\Gamma N} } \right) = {{V_{\Gamma N} } \mathord{\left/ {\vphantom {{V_{\Gamma N} } {\overline{\Gamma } }}} \right. \kern-0pt} {\overline{\Gamma } }}\overline{N},$$

$$E\left( {e_{\Gamma Z} } \right) = {{V_{\Gamma Z} } \mathord{\left/ {\vphantom {{V_{\Gamma Z} } {\overline{\Gamma } }}} \right. \kern-0pt} {\overline{\Gamma } }}\overline{Z},$$

 and

$$E\left( {e_{NZ} } \right) = {{V_{NZ} } \mathord{\left/ {\vphantom {{V_{NZ} } {\overline{N} }}} \right. \kern-0pt} {\overline{N} }}\overline{Z}.$$

 The variance and covariance terms under RSS are defined as follows:

$$V_{\Gamma }^{2} = \theta \left[ {\frac{{\sigma_{\Gamma }^{2} }}{mc} - \frac{1}{{m^{2} c}}\sum\limits_{i = 1}^{m} {\left( {\gamma_{\left( i \right)} - \overline{\Gamma } } \right)^{2} } } \right],\,V_{\rm N}^{2} = \theta \left[ {\frac{{\sigma_{\rm N}^{2} }}{mc} - \frac{1}{{m^{2} c}}\sum\limits_{i = 1}^{m} {\left( {\gamma_{\eta \left( i \right)} - \overline{\rm N} } \right)^{2} } } \right],\,$$

$$V_{\rm Z}^{2} = \theta \left[ {\frac{{\sigma_{\rm Z}^{2} }}{mc} - \frac{1}{{m^{2} c}}\sum\limits_{i = 1}^{m} {\left( {\gamma_{\zeta \left( i \right)} - \overline{\rm Z} } \right)^{2} } } \right],\,V_{{\Gamma {\rm N}}} = \theta \left[ {\frac{{\sigma_{{\Gamma {\rm N}}} }}{mc} - \frac{1}{{m^{2} c}}\sum\limits_{i = 1}^{m} {\left( {\gamma_{\left( i \right)} - \overline{\Gamma } } \right)\left( {\gamma_{\eta \left( i \right)} - \overline{\rm N} } \right)} } \right],$$

$$\,V_{{\Gamma {\rm Z}}} = \theta \left[ {\frac{{\sigma_{{\Gamma {\rm Z}}} }}{mc} - \frac{1}{{m^{2} c}}\sum\limits_{i = 1}^{m} {\left( {\gamma_{\left( i \right)} - \overline{\Gamma } } \right)\left( {\gamma_{\zeta \left( i \right)} - \overline{\rm Z} } \right)} } \right]\,V_{{{\rm N}{\rm Z}}} = \theta \left[ {\frac{{\sigma_{{{\rm N}{\rm Z}}} }}{mc} - \frac{1}{{m^{2} c}}\sum\limits_{i = 1}^{m} {\left( {\gamma_{\eta \left( i \right)} - \overline{\rm N} } \right)\left( {\gamma_{\zeta \left( i \right)} - \overline{\rm Z} } \right)} } \right],$$

 where $\theta$ is the constant for HEWMA and is given as:

$$\theta = \frac{{\left( {\omega_{E} \omega_{H} } \right)^{2} }}{{\left( {\omega_{H} - \omega_{E} } \right)^{2} }}\left[ {\frac{{\left( {1 - \omega_{E} } \right)^{2} }}{{1 - \left( {1 - \omega_{E} } \right)^{2} }} + \frac{{\left( {1 - \omega_{H} } \right)^{2} }}{{1 - \left( {1 - \omega_{H} } \right)^{2} }} - \frac{{2\left( {1 - \omega_{E} } \right)\left( {1 - \omega_{H} } \right)}}{{1 - \left( {1 - \omega_{E} } \right)\left( {1 - \omega_{H} } \right)}}} \right].$$

Substituting

$$\widehat{\gamma}_{H\Gamma \left( T \right)} = \overline{\Gamma } \left( {e_{\Gamma } + 1} \right),\,\widehat{\gamma}_{HN\left( T \right)} = \overline{N} \left( {e_{N} + 1} \right),$$

 and

$$\widehat{\gamma}_{HZ\left( T \right)} = \overline{\Gamma } \left( {e_{Z} + 1} \right)$$

into Eq. (9), the estimator can be rewritten as:

$$\widehat{\gamma}_{2 - HEW} = \overline{\Gamma } \left( {e_{\Gamma } + 1} \right)\exp \left[ {\frac{{\overline{\rm N} - \overline{N} \left( {e_{N} + 1} \right)}}{{\overline{\rm N} + \overline{N} \left( {e_{N} + 1} \right)}}} \right]\exp \left[ {\frac{{\overline{\rm Z} - \overline{\Gamma } \left( {e_{Z} + 1} \right)}}{{\overline{\rm Z} + \overline{\Gamma } \left( {e_{Z} + 1} \right)}}} \right]$$

$$= \overline{\Gamma } \left( {e_{\Gamma } + 1} \right)\exp \left( {\frac{{ - e_{N} }}{{e_{N} + 2}}} \right)\exp \left( {\frac{{ - e_{Z} }}{{e_{Z} + 2}}} \right)$$

 

$$\,\widehat{\gamma}_{2 - HEW} = \overline{\Gamma } \left( {e_{\Gamma } + 1} \right)\exp \left[ {\frac{{ - e_{N} }}{2}\left( {\frac{{e_{N} }}{2} + 1} \right)^{ - 1} } \right]\exp \left[ {\frac{{ - e_{Z} }}{2}\left( {\frac{{e_{Z} }}{2} + 1} \right)^{ - 1} } \right]$$

Using a second-order Taylor series expansion of the exponential term, the estimator can be approximated as:

$$\widehat{\gamma}_{2 - HEW} \cong \overline{\Gamma } \left( {e_{\Gamma } + 1} \right)\left[ {1 - \frac{{e_{N} }}{2}\left( {\frac{{e_{N} }}{2} + 1} \right)^{ - 1} + \frac{{e_{N}^{2} }}{8}\left( {\frac{{e_{N} }}{2} + 1} \right)^{ - 2} } \right]$$

$$\left[ {1 - \frac{{e_{Z} }}{2}\left( {\frac{{e_{Z} }}{2} + 1} \right)^{ - 1} + \frac{{e_{Z}^{2} }}{8}\left( {\frac{{e_{Z} }}{2} + 1} \right)^{ - 2} } \right]$$

 

After expanding the exponential terms and rearranging the expression, the estimator can be written as:

$$\widehat{\gamma}_{2 - HEW} \cong \overline{\Gamma } \left( {e_{\Gamma } + 1} \right)\left( {1 - \frac{{e_{N} }}{2} + \frac{{3e_{N}^{2} }}{8}} \right)\left( {1 - \frac{{e_{Z} }}{2} + \frac{{3e_{Z}^{2} }}{8}} \right)$$

Expanding the terms inside the parentheses and rearranging the expression, the estimator can be written in linear form as:

10 $$\widehat{\gamma}_{2 - HEW} \cong \overline{\Gamma } \left( {1 + e_{\Gamma } - \frac{{e_{N} }}{2} - \frac{{e_{Z} }}{2} + \frac{{3e_{Z}^{2} }}{8} + \frac{{3e_{N}^{2} }}{8} - \frac{{e_{\Gamma } e_{Z} }}{2} - \frac{{e_{\Gamma } e_{N} }}{2} + \frac{{e_{N} e_{Z} }}{4}} \right)$$

To obtain the bias, subtract $\overline{\Gamma }$ from the linear form of the estimator given in Eq. (10) and take the expected value:

$$E\left( {\widehat{\gamma}_{2 - HEW} - \overline{\Gamma } } \right) \cong \overline{\Gamma } E\left( {e_{\Gamma } - \frac{{e_{N} }}{2} - \frac{{e_{Z} }}{2} + \frac{{3e_{Z}^{2} }}{8} + \frac{{3e_{N}^{2} }}{8} - \frac{{e_{\Gamma } e_{Z} }}{2} - \frac{{e_{\Gamma } e_{N} }}{2} + \frac{{e_{N} e_{Z} }}{4}} \right)$$

$$E\left( {\widehat{\gamma}_{2 - HEW} - \overline{\Gamma } } \right) \cong \overline{\Gamma } \left\{ {E\left( {e_{\Gamma } } \right) - \frac{{E\left( {e_{N} } \right) + E\left( {e_{Z} } \right)}}{2} + \frac{{3\left[ {E\left( {e_{Z}^{2} } \right) + E\left( {e_{N}^{2} } \right)} \right]}}{8} - \frac{{E\left( {e_{\Gamma } e_{Z} } \right)}}{2} - \frac{{E\left( {e_{\Gamma } e_{N} } \right)}}{2} + \frac{{E\left( {e_{N} e_{Z} } \right)}}{4}} \right\}$$

11 $$E\left( {\widehat{\gamma}_{2 - HEW} - \overline{\Gamma } } \right) \cong \overline{\Gamma } \left\{ {\frac{{3\left[ {E\left( {e_{Z}^{2} } \right) + E\left( {e_{N}^{2} } \right)} \right]}}{8} - \frac{{E\left( {e_{\Gamma } e_{Z} } \right)}}{2} - \frac{{E\left( {e_{\Gamma } e_{N} } \right)}}{2} + \frac{{E\left( {e_{N} e_{Z} } \right)}}{4}} \right\}$$

To obtain the MSE, subtract $\overline{\Gamma }$ from the linear form of the estimator given in Eq. (10), square the resulting expression, and take the expected value.

$$E\left( {\widehat{\gamma}_{2 - HEW} - \overline{\Gamma } } \right)^{2} \cong \overline{\Gamma }^{2} E\left[ {\left( {e_{\Gamma } - \frac{{e_{N} }}{2} - \frac{{e_{Z} }}{2} + \frac{{3e_{Z}^{2} }}{8} + \frac{{3e_{N}^{2} }}{8} - \frac{{e_{\Gamma } e_{Z} }}{2} - \frac{{e_{\Gamma } e_{N} }}{2} + \frac{{e_{N} e_{Z} }}{4}} \right)^{2} } \right]$$

$$E\left( {\widehat{\gamma}_{2 - HEW} - \overline{\Gamma } } \right)^{2} \cong \overline{\Gamma }^{2} E\left[ {\left( {e_{\Gamma }^{2} + \frac{{e_{N}^{2} }}{4} + \frac{{e_{Z}^{2} }}{4} - e_{\Gamma } e_{N} - e_{\Gamma } e_{Z} + \frac{{e_{N} e_{Z} }}{4}} \right)} \right]$$

12 $$E\left( {\widehat{\gamma}_{2 - HEW} - \overline{\Gamma } } \right)^{2} \cong \overline{\Gamma }^{2} \left( {E\left( {e_{\Gamma }^{2} } \right) + \frac{{E\left( {e_{N}^{2} } \right) + E\left( {e_{Z}^{2} } \right)}}{4} - E\left( {e_{\Gamma } e_{N} } \right) - E\left( {e_{\Gamma } e_{Z} } \right) + \frac{{E\left( {e_{N} e_{Z} } \right)}}{2}} \right)$$

Substituting the expected value expressions into Eqs. (11) and (12), the bias and MSE of the estimator are obtained using a second-order approximation:

13 $$B\left( {\widehat{\gamma}_{2 - HEW} } \right) = \overline{\Gamma } \left[ {{\raise0.7ex\hbox{$3$} \!\mathord{\left/ {\vphantom {3 8}}\right.\kern-0pt} \!\lower0.7ex\hbox{$8$}}\left( {V_{\rm N}^{2} + V_{\rm Z}^{2} } \right) - {\raise0.7ex\hbox{$1$} \!\mathord{\left/ {\vphantom {1 2}}\right.\kern-0pt} \!\lower0.7ex\hbox{$2$}}\left( {V_{{\Gamma {\rm N}}} + V_{{\Gamma {\rm Z}}} } \right) + {\raise0.7ex\hbox{$1$} \!\mathord{\left/ {\vphantom {1 4}}\right.\kern-0pt} \!\lower0.7ex\hbox{$4$}}V_{{{\rm N}{\rm Z}}} } \right]$$

14 $$MSE\left( {\widehat{\gamma}_{2 - HEW} } \right) = \overline{\Gamma }^{2} \left[ {V_{\Gamma }^{2} + {\raise0.7ex\hbox{$1$} \!\mathord{\left/ {\vphantom {1 4}}\right.\kern-0pt} \!\lower0.7ex\hbox{$4$}}\left( {V_{\rm N}^{2} + V_{\rm Z}^{2} } \right) - V_{{\Gamma {\rm N}}} - V_{{\Gamma {\rm Z}}} + {\raise0.7ex\hbox{$1$} \!\mathord{\left/ {\vphantom {1 2}}\right.\kern-0pt} \!\lower0.7ex\hbox{$2$}}V_{{{\rm N}{\rm Z}}} } \right]$$

### Choosing weight parameter for proposed estimators

The selection of the weight value is crucial in memory type estimators. While EWMA involves exponential weighting of direct mean values, HEWMA represents the exponential weighting of the EWMA values. When EWMA formulas are substituted into the HEWMA formulas, it becomes evident that the weight coefficients, initially derived from the means, evolve into a more complex structure. Consequently, while EWMA typically assigns decreasing weights to past values, HEWMA does not always apply weights in a strictly decreasing order. When the HEWMA statistic is iterated four times, the coefficients are obtained as shown in Table 1.

**Table 1:** HEWMA coefficients of means for the first 4 steps.

**t**	$\widehat{\gamma}_{\gamma \left( T \right)}$	$\widehat{\gamma}_{{\gamma \left( {T - 1} \right)}}$	$\widehat{\gamma}_{{\gamma \left( {T - 2} \right)}}$	$\widehat{\gamma}_{{\gamma \left( {T - 3} \right)}}$	$\widehat{\gamma}_{{\gamma \left( {T - 4} \right)}}$
0	1	-	-	-	-
1	$1 - \omega_{E} \omega_{H}$	$\omega_{E} \omega_{H}$	-	-	-
2	$\left( {1 - \omega_{H} } \right)\left( {1 - \omega_{E} \omega_{H} } \right) + \omega_{H} \left( {1 - \omega_{E} } \right)^{2}$	$\left( {1 - \omega_{H} } \right)\omega_{E} \omega_{H} + \left( {1 - \omega_{E} } \right)\omega_{E} \omega_{H}$	$\omega_{E} \omega_{H}$	-	-
3	$$\begin{gathered} \left( {1 - \omega_{H} } \right)\left[ \begin{gathered} \left( {1 - \omega_{H} } \right)\left( {1 - \omega_{E} \omega_{H} } \right) \hfill \\ + \omega_{H} \left( {1 - \omega_{E} } \right)^{2} \hfill \\ \end{gathered} \right] \hfill \\ + \omega_{H} \left( {1 - \omega_{E} } \right)^{3} \hfill \\ \end{gathered}$$	$$\begin{gathered} \left( {1 - \omega_{H} } \right)\left[ \begin{gathered} \left( {1 - \omega_{H} } \right)\omega_{E} \omega_{H} \hfill \\ + \left( {1 - \omega_{E} } \right)\omega_{E} \omega_{H} \hfill \\ \end{gathered} \right] \hfill \\ + \omega_{E} \omega_{H} \left( {1 - \omega_{E} } \right)^{2} \hfill \\ \end{gathered}$$	$$\begin{gathered} \left( {1 - \omega_{H} } \right)\omega_{E} \omega_{H} \hfill \\ + \left( {1 - \omega_{E} } \right)\omega_{E} \omega_{H} \hfill \\ \end{gathered}$$	$\omega_{E} \omega_{H}$	-
4	$$\begin{gathered} \left( {1 - \omega_{H} } \right)\left[ \begin{gathered} \left( {1 - \omega_{H} } \right)\left[ \begin{gathered} \left( {1 - \omega_{H} } \right)\left( {1 - \omega_{E} \omega_{H} } \right) \hfill \\ + \omega_{H} \left( {1 - \omega_{E} } \right)^{2} \hfill \\ \end{gathered} \right] \hfill \\ + \omega_{H} \left( {1 - \omega_{E} } \right)^{3} \hfill \\ \end{gathered} \right] \hfill \\ + \omega_{H} \left( {1 - \omega_{E} } \right)^{4} \hfill \\ \end{gathered}$$	$$\begin{gathered} \left( {1 - \omega_{H} } \right)\left[ \begin{gathered} \left( {1 - \omega_{H} } \right)\left( {1 - \omega_{E} \omega_{H} } \right) \hfill \\ + \omega_{H} \left( {1 - \omega_{E} } \right)^{2} \hfill \\ \end{gathered} \right] \hfill \\ + \omega_{H} \left( {1 - \omega_{E} } \right)^{3} \hfill \\ \end{gathered}$$	$$\begin{gathered} \left( {1 - \omega_{H} } \right)\left[ \begin{gathered} \left( {1 - \omega_{H} } \right)\omega_{E} \omega_{H} \hfill \\ + \left( {1 - \omega_{E} } \right)\omega_{E} \omega_{H} \hfill \\ \end{gathered} \right] \hfill \\ + \omega_{E} \omega_{H} \left( {1 - \omega_{E} } \right)^{2} \hfill \\ \end{gathered}$$	$$\begin{gathered} \left( {1 - \omega_{H} } \right)\omega_{E} \omega_{H} \hfill \\ + \left( {1 - \omega_{E} } \right)\omega_{E} \omega_{H} \hfill \\ \end{gathered}$$	$\omega_{E} \omega_{H}$

When the expansion in Table 1 is extended, the general structure given in Eq. (15) emerges.

15 $$\begin{gathered} \omega_{\kappa \left( t \right)} = \omega_{E} \omega_{H} \left( {1 - \omega_{E} } \right)^{t + 1 - \kappa } \sum\limits_{j = 0}^{\kappa - 2} {\left( {1 - \omega_{H} } \right)^{j} ,\,for\,\,2 \le \kappa \le t} \hfill \\ \omega_{1\left( t \right)} = \left( {1 - \omega_{H} } \right)^{t} + \omega_{H} \left( {1 - \omega_{E} } \right)^{t} ;\,\,\,\omega_{t + 1\left( t \right)} = \omega_{E} \omega_{H} \hfill \\ \end{gathered}$$

If three or fewer past means are used, the closed-form expression in Table 1 should be applied. If four or more past means are used, the weight assigned to the past means can be pre-determined by utilizing the closed-form expression given in Eq. (15).

### Simulation study

Trivariate Normal (5,1) and trivariate Weibull(0.6,4.43) distributions were generated and used as the population. The correlation coefficients among the variables were set such that *ρ*_ΓΝ_ ≈ *ρ*_ΓΖ_ ≈ *ρ*_ΝΖ_ ≈ 0.7, 0.8, and 0.9, with a population size of 1000. In the RSS procedure, units were ranked using the first auxiliary variable (N) as a concomitant variable. RSS samples were drawn with *m* = 2, 3, and 4, and *c* = 1, 2, and 3. In accordance with previous study in the literature^22^, the weight parameters for the memory-type estimators were set at 0.5, 0.7, and 0.9. These values correspond to low, moderate, and high weighting of past observations, allowing the performance of the proposed HEWMA estimator to be evaluated under varying memory effects. RSS samples were then drawn from the generated symmetric and skewed distributions using the specified values of *m* and *c*. The estimators were computed for each sample, and this process was repeated 100,000 times. Subsequently, the Relative Efficiency (RE) values were calculated using Eq. (16). The pseudocode for the simulation procedure is provided below.

**Figure:**
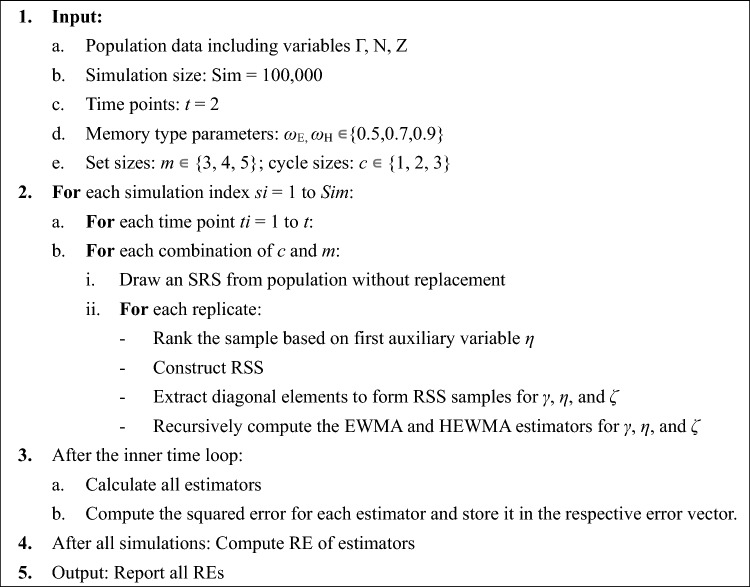
Algorithm 1 The MSE comparison of estimators.

16 $$RE_{k} = \frac{{\sum\limits_{i = 1}^{100000} {\left( {\widehat{\gamma}_{1} - \overline{\Gamma } } \right)^{2} } }}{{\sum\limits_{i = 1}^{100000} {\left( {\widehat{\gamma}_{v} - \overline{\Gamma } } \right)^{2} } }},\,k = 1 - Rat,1 - Exp,1 - {\mathrm{Re}} g,2 - Rat,2 - Exp,2 - {\mathrm{Re}} g,1 - EW,2 - HEW$$

Tables 2 and Table 3 present the RE values of estimators based on the Normal and Weibull distributions, respectively, using one and two auxiliary variables. Tables 4,5,6 report the RE values of memory-type estimators under different correlation levels for the Normal distribution, while Tables 7,8,9 present the performance of memory-type estimators under the Weibull distribution. In all tables, the maximum RE value for each group of estimators is indicated in boldface.

**Table 2:** RE values for estimators using single and two auxiliary variables at different correlations and sample sizes under Normal distribution.

*ρ*	*m*	3	4	5	3	4	5	3	4	5
	***c***	**1**	**1**	**1**	**2**	**2**	**2**	**3**	**3**	**3**
**0.7**	***RE***_**1-Rat**_	1.31908	1.27028	1.24168	1.34095	1.29202	1.24130	1.34468	1.28080	1.23056
	***RE***_**1-Exp**_	**1.42690**	1.35126	**1.30181**	1.43502	1.35887	**1.30163**	1.43454	1.35461	1.29417
	***RE***_**1-Reg**_	1.20404	**1.39500**	1.19431	**1.44951**	**1.39584**	1.23792	**1.46264**	**1.39115**	**1.32230**
	***RE***_**2-Rat**_	0.59400	0.63057	0.66212	0.62677	0.65491	0.66557	0.63041	0.65350	0.66694
	***RE***_**2-Exp**_	**1.64577**	**1.56471**	**1.52096**	**1.67419**	**1.58991**	**1.51073**	**1.66625**	**1.57795**	**1.49714**
	***RE***_**2-Reg**_	1.06321	0.46132	1.41350	0.27395	1.36329	0.85076	1.52503	1.07239	1.40948
**0.8**	***RE***_**1-Rat**_	**1.85647**	**1.69077**	**1.59468**	**1.84405**	**1.70350**	1.60074	**1.85904**	1.70883	1.61272
	***RE***_**1-Exp**_	1.67052	1.55121	1.47654	1.66622	1.55871	1.48171	1.67169	1.55985	1.48514
	***RE***_**1-Reg**_	0.65482	1.52561	1.54529	1.77758	1.69184	**1.60486**	1.84981	**1.71966**	**1.62556**
	***RE***_**2-Rat**_	0.80390	0.82988	0.85132	0.82194	0.84350	0.86325	0.84330	0.86802	0.87409
	***RE***_**2-Exp**_	**2.45005**	**2.23356**	**2.09540**	**2.43319**	**2.22906**	**2.09151**	**2.44886**	**2.25844**	**2.11758**
	***RE***_**2-Reg**_	0.28547	0.82540	0.98663	1.02341	1.11504	1.15918	1.14790	1.19809	1.20700
**0.9**	***RE***_**1-Rat**_	**3.14409**	**2.75637**	**2.54960**	**3.18944**	**2.81985**	**2.53051**	**3.20158**	**2.82778**	**2.50670**
	***RE***_**1-Exp**_	2.20279	2.03652	1.93769	2.21444	2.05098	1.92746	2.21381	2.05288	1.92281
	***RE***_**1-Reg**_	0.68508	2.37528	2.39837	2.96930	2.73152	2.48893	3.09528	2.78801	2.49712
	***RE***_**2-Rat**_	0.84193	0.87044	0.87831	0.86834	0.89170	0.89623	0.88062	0.90405	0.89286
	***RE***_**2-Exp**_	**4.03164**	**3.49413**	**3.23571**	**4.05997**	**3.57126**	**3.20586**	**4.07571**	**3.61039**	**3.16682**
	***RE***_**2-Reg**_	0.29504	0.77069	0.96678	0.98388	1.05375	1.07550	1.05532	1.10620	1.09256

**Table 3:** RE values for estimators using single and two auxiliary variables at different correlations and sample sizes under Weibull distribution.

*ρ*	*m*	3	4	5	3	4	5	3	4	5
	***c***	**1**	**1**	**1**	**2**	**2**	**2**	**3**	**3**	**3**
**0.7**	***RE***_**1-Rat**_	0.66514	0.81545	0.93631	0.94414	1.07557	1.14471	1.08308	1.17761	1.23746
	***RE***_**1-Exp**_	**1.64460**	**1.57496**	**1.56561**	**1.64861**	**1.59701**	**1.56736**	**1.64435**	**1.61032**	**1.57764**
	***RE***_**1-Reg**_	0.35608	0.63451	0.79574	0.78014	0.96243	1.06818	0.96595	1.11224	1.19977
	***RE***_**2-Rat**_	0.00267	0.06772	0.12111	0.12382	0.21522	0.30280	0.21687	0.32408	0.43180
	***RE***_**2-Exp**_	**1.56340**	**1.57357**	**1.59338**	**1.72361**	**1.72428**	**1.73337**	**1.78263**	**1.80475**	**1.83181**
	***RE***_**2-Reg**_	0.10489	0.24816	0.34235	0.38842	0.52011	0.61832	0.54149	0.66951	0.80471
**0.8**	***RE***_**1-Rat**_	0.82669	1.05749	1.14584	1.16542	1.29653	1.36280	1.35788	1.42007	1.46459
	***RE***_**1-Exp**_	**1.96460**	**1.92297**	**1.84016**	**1.97443**	**1.90607**	**1.85968**	**2.00374**	**1.92889**	**1.88253**
	***RE***_**1-Reg**_	0.33287	0.76322	0.91502	0.88878	1.07858	1.19551	1.13705	1.26643	1.35480
	***RE***_**2-Rat**_	0.01834	0.06908	0.12893	0.11520	0.19614	0.27230	0.19038	0.27662	0.34182
	***RE***_**2-Exp**_	**2.10179**	**2.18616**	**2.12571**	**2.30865**	**2.28403**	**2.28787**	**2.40957**	**2.37427**	**2.32469**
	***RE***_**2-Reg**_	0.07908	0.26419	0.36176	0.37094	0.48238	0.57493	0.50064	0.60668	0.68032
**0.9**	***RE***_**1-Rat**_	3.02353	**3.70831**	**4.05207**	**4.15764**	**4.50031**	**4.64547**	**4.62921**	**4.64367**	**4.66157**
	***RE***_**1-Exp**_	**3.39635**	3.28969	3.18022	3.22583	3.06931	2.92998	3.08392	2.89251	2.76345
	***RE***_**1-Reg**_	1.06457	2.48526	3.02204	3.03850	3.57435	3.93000	3.69082	3.94581	4.22408
	***RE***_**2-Rat**_	0.05486	0.19415	0.32779	0.26530	0.44540	0.61747	0.42287	0.59761	0.75251
	***RE***_**2-Exp**_	**5.48205**	**5.48094**	**5.54299**	**5.94506**	**5.92657**	**5.86228**	**6.23872**	**5.94373**	**5.83222**
	***RE***_**2-Reg**_	0.31966	0.62503	0.73800	0.70521	0.82287	0.91822	0.82862	0.90998	1.00505

**Table 4:** RE values of memory-type estimators under the Normal distribution with different weight parameters and sample sizes when *ρ* = 0.7.

*ω*_*E*_	*ω*_*H*_	*m*	3	4	5	3	4	5	3	4	5
		***c***	**1**	**1**	**1**	**2**	**2**	**2**	**3**	**3**	**3**
**0.5**	**-**	***RE***_**1-EW**_	2.68412	2.54443	2.49319	2.66315	2.60591	2.49647	2.70492	2.56804	2.48110
	**0.5**	***RE***_**2-HEW**_	3.34908	3.11532	**3.03748**	3.33401	3.18785	**3.04528**	**3.36186**	3.16691	3.03070
	**0.7**	***RE***_**2-HEW**_	2.85117	2.70664	2.58139	2.92550	2.73405	2.60334	2.91612	2.69620	2.61199
	**0.9**	***RE***_**2-HEW**_	2.02466	1.90156	1.84098	2.01806	1.93734	1.85108	2.05896	1.94767	1.84790
**0.7**	**-**	***RE***_**1-EW**_	2.31199	2.19703	2.12411	2.29780	2.22155	2.14576	2.32727	2.22482	2.15342
	**0.5**	***RE***_**2-HEW**_	3.32145	3.12671	3.01071	3.30655	3.16001	3.03984	3.34270	3.18198	3.02212
	**0.7**	***RE***_**2-HEW**_	2.87455	2.71236	2.61159	2.87432	2.73071	2.61516	2.91110	2.76307	2.61686
	**0.9**	***RE***_**2-HEW**_	2.01813	1.91863	1.83919	2.02696	1.92632	1.85228	2.04673	1.93297	1.86248
**0.9**	**-**	***RE***_**1-EW**_	1.63719	1.56561	1.50011	1.63908	1.58005	1.51206	1.63571	1.59224	1.52718
	**0.5**	***RE***_**2-HEW**_	**3.36596**	**3.15508**	3.00954	**3.36284**	**3.19022**	3.01541	3.32235	**3.18225**	**3.04648**
	**0.7**	***RE***_**2-HEW**_	2.86823	2.70866	2.62280	2.88722	2.72381	2.61489	2.85127	2.70151	2.62117
	**0.9**	***RE***_**2-HEW**_	2.03154	1.90995	1.85616	2.04992	1.92873	1.83393	2.04098	1.93027	1.85911

**Table 5:** RE values of memory-type estimators under the Normal distribution with different weight parameters and sample sizes when *ρ* = 0.8.

*ω*_*E*_	*ω*_*H*_	*m*	3	4	5	3	4	5	3	4	5
		***c***	**1**	**1**	**1**	**2**	**2**	**2**	**3**	**3**	**3**
**0.5**	**-**	***RE***_**1-EW**_	3.75980	3.41033	3.19737	3.68207	3.41563	3.22420	3.71439	3.43282	3.22215
	**0.5**	***RE***_**2-HEW**_	4.94659	**4.50866**	4.18141	4.87244	4.48620	4.19487	4.91253	**4.53112**	**4.23766**
	**0.7**	***RE***_**2-HEW**_	4.19877	3.87010	3.62891	4.27112	3.89845	3.64755	4.26690	3.87198	3.67097
	**0.9**	***RE***_**2-HEW**_	2.97682	2.72871	2.57291	2.99906	2.72511	2.54597	2.98721	2.74162	2.55927
**0.7**	**-**	***RE***_**1-EW**_	3.19606	2.91339	2.76857	3.21711	2.96909	2.75893	3.24272	2.94924	2.76954
	**0.5**	***RE***_**2-HEW**_	4.94301	4.45049	**4.21037**	4.95446	**4.57285**	4.22781	4.94284	4.51450	4.19528
	**0.7**	***RE***_**2-HEW**_	4.26537	3.89434	3.61988	4.25563	3.93091	3.58974	4.27198	3.91001	3.62848
	**0.9**	***RE***_**2-HEW**_	2.97706	2.71265	2.55199	3.01471	2.71767	2.54836	3.01677	2.75449	2.54500
**0.9**	**-**	***RE***_**1-EW**_	2.26970	2.07978	1.93697	2.26957	2.08832	1.95280	2.28902	2.08618	1.95068
	**0.5**	***RE***_**2-HEW**_	**4.96202**	4.47340	4.16653	**4.96819**	4.49382	**4.23360**	**4.95808**	4.49855	4.19867
	**0.7**	***RE***_**2-HEW**_	4.26967	3.83038	3.60028	4.24673	3.85553	3.60292	4.24636	3.88428	3.61124
	**0.9**	***RE***_**2-HEW**_	2.95797	2.71750	2.53479	3.00797	2.72315	2.53249	3.00833	2.75436	2.57068

**Table 6:** RE values of memory-type estimators under the Normal distribution with different weight parameters and sample sizes when *ρ* = 0.9.

*ω*_*E*_	*ω*_*H*_	*m*	3	4	5	3	4	5	3	4	5
		***c***	**1**	**1**	**1**	**2**	**2**	**2**	**3**	**3**	**3**
**0.5**	**-**	***RE***_**1-EW**_	6.36389	5.53806	5.09285	6.40910	5.63012	5.06278	6.39880	5.68559	5.02990
	**0.5**	***RE***_**2-HEW**_	8.11574	7.00653	**6.46128**	8.14019	**7.14245**	6.40383	8.11236	**7.22048**	6.36203
	**0.7**	***RE***_**2-HEW**_	6.98061	6.07391	5.56764	7.14124	6.07723	5.47528	7.01424	6.12994	5.54002
	**0.9**	***RE***_**2-HEW**_	4.90033	4.32980	3.91072	4.97248	4.32532	3.92019	5.00127	4.35035	3.93537
**0.7**	**-**	***RE***_**1-EW**_	5.50135	4.83278	4.34947	5.57477	4.83131	4.37842	5.51932	4.87147	4.32995
	**0.5**	***RE***_**2-HEW**_	**8.14013**	**7.09366**	6.42356	8.14920	7.10764	6.35367	8.14633	7.14594	6.35408
	**0.7**	***RE***_**2-HEW**_	7.04665	6.08261	5.54188	7.06889	6.16470	6.35367	7.05383	6.14964	5.56625
	**0.9**	***RE***_**2-HEW**_	4.92488	4.41620	3.92591	4.88622	4.35168	3.90030	4.97779	4.30967	3.87326
**0.9**	**-**	***RE***_**1-EW**_	3.85563	3.39228	3.08598	3.95366	3.41446	3.09455	3.91061	3.42919	3.06472
	**0.5**	***RE***_**2-HEW**_	8.05143	7.05752	6.45433	**8.20294**	7.13378	**6.42272**	**8.14951**	7.14413	**6.40230**
	**0.7**	***RE***_**2-HEW**_	7.07261	6.14140	5.50965	7.04067	6.11734	5.53601	7.00106	6.17987	5.57311
	**0.9**	***RE***_**2-HEW**_	4.91374	4.35155	3.88092	4.97638	4.34228	3.91115	4.98888	4.35499	3.90902

**Table 7:** RE values of memory-type estimators under the Weibull distribution with different weight parameters and sample sizes when* ρ* = 0.7.

*ω*_*E*_	*ω*_*H*_	*m*	3	4	5	3	4	5	3	4	5
		***c***	**1**	**1**	**1**	**2**	**2**	**2**	**3**	**3**	**3**
**0.5**	**-**	***RE***_**1-EW**_	1.92030	2.12600	2.24936	2.33204	2.47560	2.54739	2.52145	2.62471	2.70845
	**0.5**	***RE***_**2-HEW**_	3.41013	3.47360	**3.44714**	**3.71371**	3.65922	3.64528	**3.78798**	3.77426	**3.82583**
	**0.7**	***RE***_**2-HEW**_	2.96450	3.05924	3.04291	3.18429	3.24406	3.15279	3.34482	3.24899	3.18206
	**0.9**	***RE***_**2-HEW**_	2.12867	2.12173	2.10253	2.16935	2.23372	2.26460	2.30661	2.25983	2.26913
**0.7**	**-**	***RE***_**1-EW**_	1.61676	1.87553	1.94880	2.02994	2.14617	2.26296	2.15964	2.28970	2.88752
	**0.5**	***RE***_**2-HEW**_	3.35689	3.44979	3.40652	3.65343	3.62099	**3.73786**	3.75721	**3.82309**	3.72322
	**0.7**	***RE***_**2-HEW**_	3.09687	3.00583	3.10925	3.22282	3.29489	3.17764	3.30306	3.31157	3.17255
	**0.9**	***RE***_**2-HEW**_	2.08003	2.14014	2.13013	2.22608	2.24816	2.21726	2.29385	2.24058	2.23743
**0.9**	**-**	***RE***_**1-EW**_	1.07926	1.24238	1.33598	1.34671	1.43957	1.50135	1.46399	1.51324	1.53757
	**0.5**	***RE***_**2-HEW**_	**3.43122**	**3.50409**	3.43064	3.66559	**3.70096**	3.64152	3.72533	3.70431	3.66066
	**0.7**	***RE***_**2-HEW**_	2.93538	3.08233	3.03603	3.23423	3.20343	3.19601	3.29176	3.30131	3.26016
	**0.9**	***RE***_**2-HEW**_	2.21284	2.16038	2.08765	2.22748	2.20925	2.19093	2.31566	2.27365	2.23275

**Table 8:** RE values of memory-type estimators under the Weibull distribution with different weight parameters and sample sizes when* ρ* = 0.8.

*ω*_*E*_	*ω*_*H*_	*m*	3	4	5	3	4	5	3	4	5
		***c***	**1**	**1**	**1**	**2**	**2**	**2**	**3**	**3**	**3**
**0.5**	**-**	***RE***_**1-EW**_	2.34854	2.65828	2.73170	2.84290	2.97280	3.04384	3.11254	3.12830	3.18152
	**0.5**	***RE***_**2-HEW**_	4.52956	**4.64824**	**4.55138**	4.89599	4.81916	**4.81556**	5.11695	4.95398	4.87441
	**0.7**	***RE***_**2-HEW**_	4.09327	4.11737	4.09020	4.32206	4.27740	4.14506	4.48438	4.33327	4.18529
	**0.9**	***RE***_**2-HEW**_	2.91697	2.85018	2.79284	3.01716	3.00594	2.87713	3.06281	3.00197	2.98127
**0.7**	**-**	***RE***_**1-EW**_	2.07111	2.31095	2.35925	2.50794	2.57918	2.64443	2.68392	2.75134	2.77195
	**0.5**	***RE***_**2-HEW**_	4.58526	4.53854	4.50813	4.87142	4.80313	4.78084	5.03089	5.00249	**4.92777**
	**0.7**	***RE***_**2-HEW**_	4.08573	4.08325	4.01345	4.38575	4.21352	4.16633	4.50791	4.30588	4.26043
	**0.9**	***RE***_**2-HEW**_	2.92380	2.91056	2.82423	3.00461	2.96771	2.87539	3.11628	3.03424	2.96548
**0.9**	**-**	***RE***_**1-EW**_	1.37264	1.53999	1.62935	1.67609	1.77153	1.79275	1.85417	1.89750	1.91780
	**0.5**	***RE***_**2-HEW**_	**4.62761**	4.55227	4.55105	**4.91752**	**4.86511**	4.76210	**5.17995**	**5.07348**	4.92416
	**0.7**	***RE***_**2-HEW**_	4.17284	3.98035	4.01313	4.30672	4.22525	4.21086	4.43650	4.38420	4.28121
	**0.9**	***RE***_**2-HEW**_	2.86877	2.79514	2.76780	3.00418	2.98478	2.89218	3.09565	3.02002	2.95416

**Table 9:** RE values of memory-type estimators under the Weibull distribution with different weight parameters and sample sizes when* ρ* = 0.9.

*ω*_*E*_	*ω*_*H*_	*m*	3	4	5	3	4	5	3	4	5
		***c***	**1**	**1**	**1**	**2**	**2**	**2**	**3**	**3**	**3**
**0.5**	**-**	***RE***_**1-EW**_	6.36389	5.53806	5.09285	6.40910	5.63012	5.06278	6.39880	5.68559	5.02990
	**0.5**	***RE***_**2-HEW**_	8.11574	7.00653	**6.46128**	8.14019	**7.14245**	6.40383	8.11236	**7.22048**	6.36203
	**0.7**	***RE***_**2-HEW**_	6.98061	6.07391	5.56764	7.14124	6.07723	5.47528	7.01424	6.12994	5.54002
	**0.9**	***RE***_**2-HEW**_	4.90033	4.32980	3.91072	4.97248	4.32532	3.92019	5.00127	4.35035	3.93537
**0.7**	**-**	***RE***_**1-EW**_	5.50135	4.83278	4.34947	5.57477	4.83131	4.37842	5.51932	4.87147	4.32995
	**0.5**	***RE***_**2-HEW**_	**8.14013**	**7.09366**	6.42356	8.14920	7.10764	6.35367	8.14633	7.14594	6.35408
	**0.7**	***RE***_**2-HEW**_	7.04665	6.08261	5.54188	7.06889	6.16470	6.35367	7.05383	6.14964	5.56625
	**0.9**	***RE***_**2-HEW**_	4.92488	4.41620	3.92591	4.88622	4.35168	3.90030	4.97779	4.30967	3.87326
**0.9**	**-**	***RE***_**1-EW**_	8.14082	8.81880	9.25370	9.58042	9.66189	9.53167	10.03323	9.49085	9.20088
	**0.5**	***RE***_**2-HEW**_	11.60983	11.56432	**11.71737**	**12.61884**	12.22608	**12.04552**	**12.82516**	**12.09089**	**11.61112**
	**0.7**	***RE***_**2-HEW**_	10.35988	10.15692	10.04954	10.89513	10.35618	10.10542	11.27980	10.74029	10.29075
	**0.9**	***RE***_**2-HEW**_	7.26130	7.00548	6.99161	7.65063	7.44675	7.31005	7.77976	7.52101	7.27556

## Real data based simulation

This section applies the simulation study to real datasets instead of synthetic ones derived from a distribution. The real datasets used as populations were obtained from the CDC^40^. This dataset was selected because it provides a real-world example where auxiliary variables are available and can be effectively utilized within the RSS framework. In these datasets, causes of death across the 51 states of the USA in 2022 and 2023 are classified according to ICD-10 (International Classification of Diseases, 10th Revision) codes. Consistent with the simulation study, the set sizes *m* = 3, 4, and 5 and the cycles *c* = 1 and 2 were used. Since *t* = 2 was selected, the current mean corresponds to data from 2023, and the past mean to 2022.

The first real dataset covers the number of deaths from tobacco-related thoracic and upper aerodigestive tract cancers in the 51 states of the USA in 2022 and 2023. The study variable (Γ) is the number of deaths from C34 (malignant neoplasm of the bronchus and lung) in 2023. The first auxiliary variable (Ν) is the number of deaths from C15 (malignant neoplasm of the esophagus) in 2023, and the second auxiliary variable (Ζ) corresponds to deaths from C32 (malignant neoplasm of the larynx) in the same year. Unlike the synthetic datasets and the second real dataset, the first dataset contains "0" values, as there were no deaths from the specified diseases in certain states during the given years. The second dataset covers the number of deaths from alcohol-related liver diseases and disorders in the same states and years. The study variable (Γ) is the number of deaths from C22 (malignant neoplasm of the liver and intrahepatic bile ducts- malignant liver cancer). The first auxiliary variable (Ν) is the number of deaths from F10 (mental and behavioural disorders due to use of alcohol), and the second auxiliary variable (Ζ) corresponds to deaths from K70 (alcoholic liver disease).

**Fig. 1:**
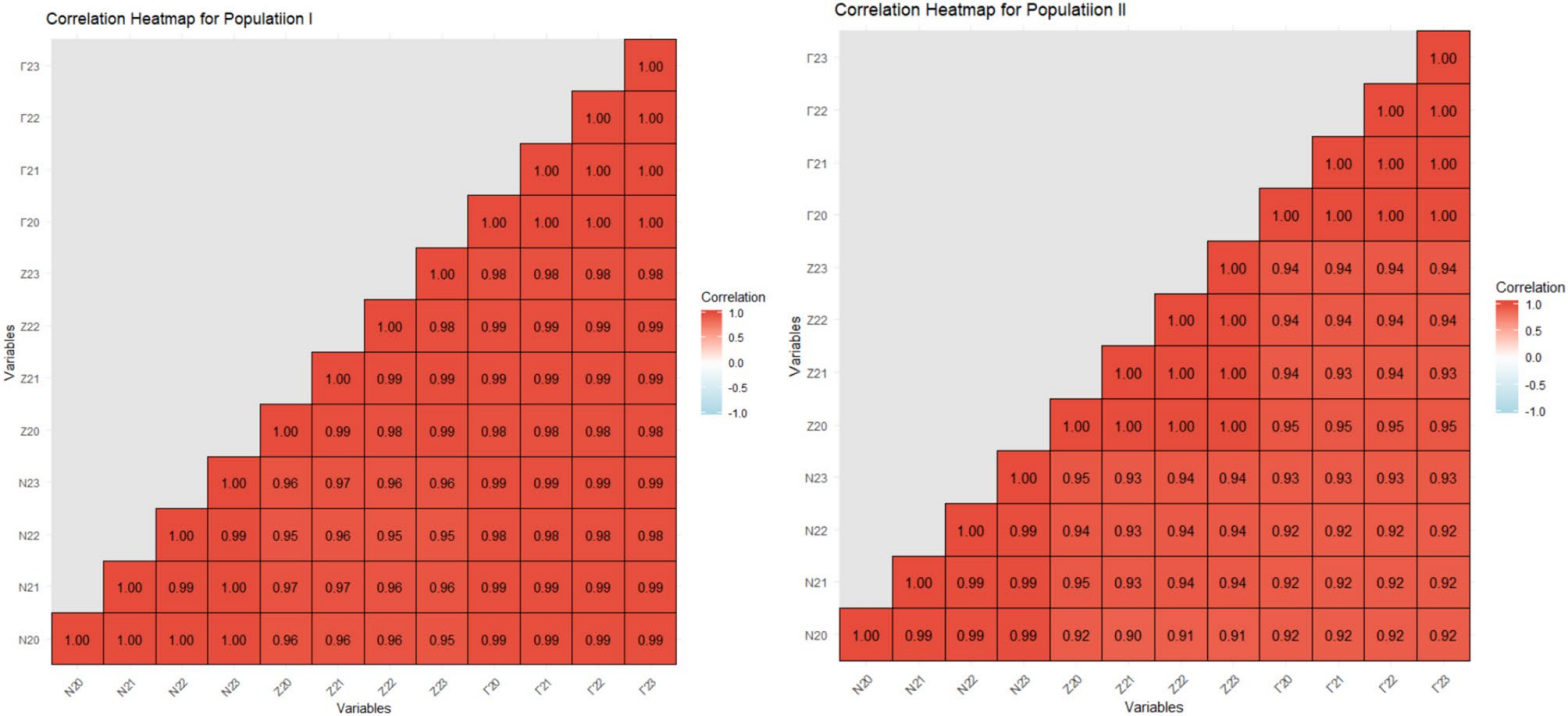
Correlation heat maps of the study and auxiliary variables for the two real populations.

Table 10 summarizes these two populations. Figure 1 presents a heat map illustrating the correlations among variables for both datasets by year, revealing a high degree of correlation. The heatmaps indicate strong positive correlations between the auxiliary variables and the study variable, supporting the applicability of RSS and the HEWMA-based estimator in these datasets. The RE results for the real datasets are presented in Table 11.

**Table 10:** Population parameters for real data sets.

Population I (Tobacco-related data)	Variables	Min–Max	Mean ± SD	Skewness- Kurtosis
	Γ_2023_	147–10,489	2572.74510** ± **2464.79735	1.50879–1.94302
	Ν_2023_	13–1293	309.54902** ± **297.96653	1.58358–2.03649
	Ζ_2023_	0–345	70.03922** ± **77.30122	1.54477–2.16771
	Γ_2022_	172–10,401	2578.60784** ± **2461.46612	1.48729–1.85339
	Ν_2022_	18–1396	309.45098** ± **299.47129	1.70227–2.70419
	Ζ_2022_	0–320	70.41176** ± **75.65875	1.3951–1.32722
Population II (Alcohol-related data)	Variables	Min–Max	Mean ± SD	Skewness- Kurtosis
	Γ_2023_	39–3746	609.9412 ± 710.0618	2.62498–7.68694
	Ν_2023_	20–1491	299.6863 ± 286.4539	1.86983–4.47458
	Ζ_2023_	32–4479	556.1176 ± 702.0378	3.72366–16.97573
	Γ_2022_	48–3704	588.4706 ± 691.9754	2.67268–8.06463
	Ν_2022_	29–1584	312.1569 ± 291.5197	1.94695–5.26192
	Ζ_2022_	27–4662	601.5882 ± 731.5328	3.63520–16.42651

**Table 11:** RE values of estimators for real data sets.

Population I		*m*	3	4	5	3	4	5
		***c***	**1**	**1**	**1**	**2**	**2**	**2**
		***RE***_**1-Rat**_	14.90723	13.70893	12.21484	17.65715	15.33353	13.20041
		***RE***_**1-Exp**_	3.47050	3.43146	3.39309	3.55591	3.50280	3.43367
		***RE***_**1-Reg**_	3.57977	9.02204	10.44519	14.01468	13.85812	12.56719
		***RE***_**2-Rat**_	N/A	0.33389	0.43821	0.37977	0.48543	0.55371
		***RE***_**2-Exp**_	**29.07717**	**25.69244**	**22.14757**	**31.17411**	**26.73922**	**22.62840**
		***RE***_**2-Reg**_	N/A	0.87424	0.94671	0.94635	0.97965	0.99278
***ω***_***E***_	***ω***_***H***_	***m***	**3**	**4**	**5**	**3**	**4**	**5**
		***c***	**1**	**1**	**1**	**2**	**2**	**2**
**0.5**	**-**	***RE***_**1-EW**_	13.71790	9.99537	7.80187	3.13118	3.57208	3.81753
	**0.5**	***RE***_**2-HEW**_	31.32303	23.59471	17.93410	6.24299	7.02919	7.63235
	**0.7**	***RE***_**2-HEW**_	38.52286	28.74152	21.90625	8.23789	9.21016	9.82791
	**0.9**	***RE***_**2-HEW**_	44.65046	33.49899	26.59516	11.01982	12.21714	12.91601
**0.7**	**-**	***RE***_**1-EW**_	19.55456	15.54396	12.79940	7.29058	7.90519	8.03076
	**0.5**	***RE***_**2-HEW**_	38.00840	28.62300	21.92003	8.17260	9.15099	9.88894
	**0.7**	***RE***_**2-HEW**_	46.55271	35.79911	28.42969	12.44749	13.57558	14.45853
	**0.9**	***RE***_**2-HEW**_	**49.55840**	40.09309	32.37032	19.67104	20.71154	20.75844
**0.9**	**-**	***RE***_**1-EW**_	18.84048	16.72827	14.37684	17.34787	15.93403	14.09909
	**0.5**	***RE***_**2-HEW**_	44.08468	33.97245	26.43600	11.12650	12.30301	12.89388
	**0.7**	***RE***_**2-HEW**_	49.36723	**40.53539**	**32.51156**	19.44110	20.35254	20.86930
	**0.9**	***RE***_**2-HEW**_	43.03968	36.77969	30.99161	**31.85561**	**30.14087**	**27.47612**
**Population II**		***m***	**3**	**4**	**5**	**3**	**4**	**5**
		***c***	**1**	**1**	**1**	**2**	**2**	**2**
		***RE***_**1-Rat**_	4.86006	4.17360	3.68465	4.59618	4.01514	3.61486
		***RE***_**1-Exp**_	2.37675	2.15408	2.01529	2.21310	2.05957	1.95286
		***RE***_**1-Reg**_	0.70917	3.18848	3.64719	4.30887	3.86424	3.47444
		***RE***_**2-Rat**_	0.80803	1.13285	1.31456	1.10299	1.27234	1.31129
		***RE***_**2-Exp**_	**6.51931**	**5.74353**	**5.18053**	**6.47906**	**5.81492**	**5.29642**
		***RE***_**2-Reg**_	0.31286	0.97140	1.21015	1.13775	1.24452	1.24113
***ω***_***E***_	***ω***_***H***_	***m***	**3**	**4**	**5**	**3**	**4**	**5**
		***c***	**1**	**1**	**1**	**2**	**2**	**2**
**0.5**	**-**	***RE***_**1-EW**_	7.01923	5.26884	4.46557	3.60845	3.67459	3.55989
	**0.5**	***RE***_**2-HEW**_	8.62873	6.87490	6.03633	5.53713	5.34538	4.97095
	**0.7**	***RE***_**2-HEW**_	9.94339	8.16074	7.15666	6.75644	6.50454	6.05886
	**0.9**	***RE***_**2-HEW**_	11.08743	9.19370	8.20925	8.07794	7.74472	7.11500
**0.7**	**-**	***RE***_**1-EW**_	7.32332	6.00113	5.21867	5.39208	5.01763	4.68745
	**0.5**	***RE***_**2-HEW**_	9.83970	8.16048	7.17681	6.86663	6.44785	6.07526
	**0.7**	***RE***_**2-HEW**_	10.79940	9.71893	8.55448	8.49112	8.13541	7.50146
	**0.9**	***RE***_**2-HEW**_	**11.35859**	**9.89203**	**8.91138**	9.64975	**9.08794**	8.29260
**0.9**	**-**	***RE***_**1-EW**_	5.91709	5.06571	4.52047	5.40823	4.79584	4.32694
	**0.5**	***RE***_**2-HEW**_	10.90374	9.33807	8.26392	7.99854	7.68712	7.25445
	**0.7**	***RE***_**2-HEW**_	11.24871	9.79681	8.82313	**9.67863**	9.07094	**8.31927**
	**0.9**	***RE***_**2-HEW**_	9.49364	8.41236	7.61668	9.06070	8.18529	7.44548

## Discussion

Under the Normal distribution with a correlation of 0.7, estimators using a single auxiliary variable achieved a maximum RE of 1.46, while those using two auxiliary variables reached an RE of 1.67. EWMA-based memory type estimators attained an RE as high as 2.705 (with *ω*_E_ = 0.5, *m* = *c* = 3). In comparison, the estimator introduced in this study reached an RE of 3.36 under the same correlation (*ω*_E_ = 0.9, *ω*_H_ = 0.5, *m* = 3, *c* = 1). This estimator also demonstrated strong performance across all examined sample sizes for this correlation level. Similar patterns were observed at correlation levels of 0.8 and 0.9, with consistent improvements noted across various distributional scenarios, including the right-skewed Weibull distribution. These results clearly demonstrate that incorporating both memory effects and multiple auxiliary variables leads to substantial gains in estimation efficiency, particularly under moderate to high correlation structures.

The real-world datasets used in this study differ from the simulated populations in several key aspects: they have a smaller size (51 units each), the first dataset includes zero values, and both exhibit relatively higher correlations between variables. These characteristics posed challenges for traditional ratio and regression-type estimators, particularly due to the presence of zeros in the Z variable of the first real dataset, which can render such computations undefined. In contrast, the proposed estimator maintained strong performance, achieving higher RE in the first real dataset compared to both the second real dataset and the synthetic datasets. This improvement can be attributed to its ability to smooth out extreme values and effectively incorporate past information, offering robustness where classical estimators tend to struggle.

In the simulation studies, the best results for the proposed estimator were generally observed when *ω*_H_ was fixed at 0.5, regardless of the *ω*_E_ value. In contrast, analysis of the real data sets indicated improved outcomes when both *ω*_E_ and *ω*_H_ were set to higher values (0.7 or 0.9). This difference may be related to the smaller sample size, stronger correlations, and potentially more stable temporal structure in the real data, which could increase the relevance of past information when greater weight is assigned. As discussed in Sect. 2.2, the weight parameters used in the HEWMA estimator are more complex than in the traditional EWMA method. Since the choice of weight parameters, especially for *ω*_E_ and *ω*_H_, affects the performance of the estimator under different conditions, this warrants further investigation. Furthermore, the RE value of the proposed estimator decreases as the set size increases, and increases as the number of cycles increases.

Compared to existing RSS-based estimators, the proposed HEWMA estimator not only incorporates dual auxiliary variables but also effectively utilizes temporal information, which explains its consistently superior performance over existing estimators across all examined scenarios. This indicates that integrating memory structures with multiple auxiliary variables provides a systematic advantage in improving estimation efficiency within the RSS framework.

While the results demonstrate improved efficiency of the proposed estimator under the considered scenarios, the findings should be interpreted in light of certain limitations. The simulation study was conducted under specific distributional assumptions and parameter settings, and the real-data application was based on a limited number of datasets. Therefore, the observed performance advantages may vary under different sampling conditions, correlation structures, or distributional forms. In particular, the sensitivity of the estimator to different choices of weight parameters and sampling designs remains an open area for future research. Further empirical studies using additional datasets and alternative population structures would help to provide a broader evaluation of the estimator’s practical performance.

## Conclusion

This study examined the performance of a HEWMA-based memory type estimator that incorporates two auxiliary variables within the RSS scheme. Mathematical expressions for the estimator’s bias and MSE were derived, and its performance was evaluated through a series of simulation experiments and real-world mortality datasets. The results demonstrate that incorporating HEWMA statistics into the RSS framework can substantially improve estimation efficiency relative to existing estimators. The findings across different conditions, such as varying sample sizes, distributional shapes, and levels of correlation, including zero values, suggest that the estimator performs competitively, with relatively high efficiency and stable results.

Despite these encouraging results, the proposed approach is subject to certain limitations. In particular, the performance evaluation was conducted under selected simulation settings and specific real datasets. In practical applications, factors such as ranking errors, measurement errors in auxiliary variables, or different correlation structures may influence the estimator’s efficiency. These aspects should be carefully considered when applying the estimator in practice.

For future research, this estimator could be extended to alternative sampling strategies, including stratified or systematic sampling. Additionally, the impact of ranking inaccuracies or measurement errors in auxiliary variables remains an open area for investigation. The incorporation of machine learning approaches for ranking or variable selection may also enhance the estimator’s utility in more complex or data-limited environments. Given its overall performance, this estimator may be particularly useful in practical applications requiring efficient estimation from small or structured samples, such as public health surveillance or environmental monitoring. Future work may also explore the development of new estimators that integrate HEWMA, EWMA, and HEEWMA approaches with multiple auxiliary variables, along with a more comprehensive theoretical comparison with recently proposed memory-type estimators. In addition, adaptive selection of HEWMA weight parameters and investigation of performance under different distributional assumptions or sampling designs could further clarify the conditions under which the proposed estimator provides the greatest benefit and highlight potential directions for methodological improvements.

## Supplementary Information

Supplementary Information 1.

Supplementary Information 2.

## Data Availability

All data supporting the findings of this study are included within the manuscript and its supplementary files.
